# Attitudes and Career Preferences Towards Psychiatry in South Asia: A Narrative Review of Medical Student and Physician Perspectives

**DOI:** 10.1002/hsr2.73060

**Published:** 2026-08-18

**Authors:** S. M. Yasir Arafat, Sadeed Hossain

**Affiliations:** ^1^ Biomedical Research Foundation Dhaka Bangladesh; ^2^ Department of Psychiatry Bangladesh Specialized Hospital Dhaka Bangladesh

**Keywords:** attitude towards psychiatry, medical students, mental health, physicians, psychiatry, South Asia

## Abstract

**Background and Aims:**

The role of psychiatry in medical science has been debated, sometimes questioned, and not always fully respected compared to other medical fields. However, the attitude towards psychiatry has been poorly discussed in South Asia. We aimed to examine the attitudes of medical students and physicians toward psychiatry in South Asia and to identify factors influencing career preference toward psychiatry.

**Methods:**

A narrative review was conducted, covering eight South Asian countries: Afghanistan, Bangladesh, Bhutan, India, Maldives, Nepal, Pakistan, and Sri Lanka. Peer‐reviewed English articles published from inception to search date about attitudes, perceptions, or career preferences in psychiatry among medical students and physicians were included. Attitudes were analyzed descriptively, with special focus on the impact of psychiatry exposure and gender differences.

**Results:**

The search identified 58 studies. Most studies (*n* = 41) reported positive attitudes toward psychiatry, though career preference remained low. Exposure to psychiatry by clerkships, clinical rotations, or lectures improved attitudes and career preferences in 20 of 28 studies. Female students consistently showed more interest in psychiatry than male students. Attitudes became more favorable in recent studies. Despite improvements in perception, psychiatry remained one of the least preferred specialties, with career choice typically ranging from 5% to 25%.

**Conclusions:**

Attitudes toward psychiatry among medical students and physicians in South Asia are generally positive; however, career preference remains low. Early and repeated psychiatry exposure, mentorship, and curricular reforms are recommended to increase recruitment.

## 1 Introduction

South Asia consists of eight low‐ and middle‐income countries (LMICs) namely, Afghanistan, Bangladesh, Bhutan, India, Maldives, Nepal, Pakistan, and Sri Lanka [1]. The region contains about 2 billion people, making up nearly one‐fourth of the world's population. It is a diverse area with many ethnicities, cultures, languages, religions, and political systems, ranging from Islamic republics to parliamentary democracies and constitutional monarchies [1, 2, 3]. Despite recent improvements in healthcare, South Asia continues to face challenges such as poverty, fragile health systems, political instability, natural disasters, and conflict, all of which have increased the burden of mental disorders [2]. Many people are suffering from various mental health disorders due to rapid demographic and lifestyle changes, around one‐fifth of psychiatrically ill patients in the world live in this region [4]. Nonetheless, policies on mental disorders have not gotten priority [5], and the majority of South Asian countries still allocate less than 1% of their health budgets to mental health, and they have less than one psychiatrist per 100,000 population [6, 7]. Additionally, there is high stigma, low mental health literacy, super‐natural attribution of psychiatric symptoms in the region resulting in high services gap. There is a lack of mental health insurance in the region which results in a high out‐of‐pocket expense for the treatment of psychiatric disorders [2].

The role of psychiatry in medical science has been debated, sometimes questioned, and not always fully respected compared to other medical fields. It has often been viewed as less scientific, less prestigious, or less rewarding compared with other specialties [8]. These negative perceptions towards psychiatry may influence the choice of career of a medical student or physician. Many students do not choose psychiatry as a career due to a lack of respect, low salary, and negative comments by friends, colleagues, and family about choosing psychiatry [9, 10]. Studies have reported that after exposure to psychiatry, an overall positive attitudinal change had developed [11, 12]; however, students are still not interested in choosing psychiatry as a career [8]. Understanding factors that influence medical students' and practicing physicians' preference towards psychiatry as a career is therefore necessary to modify and improve recruitment and mentoring strategies that will increase the preference of students choosing psychiatry.

Studies across South Asia have examined the perspectives of medical students and physicians on psychiatry in terms of its scientific legitimacy, therapeutic effectiveness, career prospects, and social value. While some studies found gradual improvements in attitudes [13, 14], others highlight persistent challenges such as stigma, misconceptions, and a lack of interest in psychiatry as a career choice [15, 16]. One previous systematic review was conducted assessing attitudes of medical students and found a positive attitude with a lower career preference [8]. This narrative review aimed to examine the attitudes of medical students and physicians toward psychiatry in South Asia, highlighting regional patterns within a global perspective as well as the trend over time. It included the papers published after the previous review as well as papers assessing the concepts among physicians (includes psychiatry residents/postgraduate psychiatry training). The variable findings would be valuable to understand regional trends and consider measures for increasing the attitudes and career preferences in the region.

## 2 Methods

### 2.1 Search Strategy

A web‐based search was conducted without any time restrictions on publication date. The search was performed in PubMed, Scopus, and Google Scholar using both Medical Subject Headings (MeSH) and free‐text terms to ensure sensitivity and relevance on August 02, 2025. Boolean operators were applied to formulate the search string:

(“Attitude” OR “Perception” OR “Career choice” OR “Career preference” OR “Specialty choice” OR “Specialty preference” OR “Interest in psychiatry”)

AND

(“Psychiatry” OR “Psychiatric specialty”)

AND

(“Medical students” OR “MBBS students” OR “Medical undergraduates” OR “Intern physicians” OR “Physicians” OR “Physicians” OR “Medical graduates”)

AND

(“South Asia” OR “Afghanistan” OR “Bangladesh” OR “Bhutan” OR “India” OR “Maldives” OR “Nepal” OR “Pakistan” OR “Sri Lanka”)

### 2.2 Inclusion Criteria

Research articles eligible for inclusion were original studies and quantitative analyses available in full‐text form. Only peer‐reviewed articles published in English from inception to search date were included. The study was included if it focused on attitude, perception, career choice or preference in psychiatry that occurred in these eight South Asian countries.

### 2.3 Exclusion Criteria

Studies were excluded if they were review articles, editorials, opinion pieces, qualitative studies, multiple reports published from the same data, or reports based solely on media coverage. In addition, studies on migrant populations residing outside the South Asian region were excluded to ensure geographic specificity. Duplicate records were removed, and all retrieved studies were manually screened for relevance based on titles, abstracts, and full texts.

### 2.4 Outcome Variables

From each included study, the following variables were extracted:

study, publication year, country, sample size, tools/instruments used, and key findings. All extracted data were analyzed descriptively using Microsoft Excel.

### 2.5 Study Design

This work was conducted as a narrative review, using a descriptive synthesis of published papers. A formal meta‐analysis was not done because the included studies used heterogeneous and often non‐standardized instruments to assess attitudes and perceptions toward psychiatry. The terms *“psychiatric posting,” “clinical posting,”* and *“psychiatry clinical rotation”* refer to the same form of clinical training in psychiatry and are used interchangeably in this narrative review to preserve consistency with the terminology of the included studies.

### 2.6 Article Selection and Data Extraction

For this narrative review, study selection was performed by one author applying the pre‐specified inclusion and exclusion criteria, with all borderline or uncertain inclusion decisions discussed and jointly resolved with the lead author. Hand search was conducted from previous articles to identify papers satisfying the selection criteria. One author extracted the outcome variables which was checked by the lead author.

Studies published in 2025 were included those had their online‐first or ahead‐of‐print version available on or before the search date of August 2, 2025; formal print/issue publication may postdate the search date.

This review was reported in accordance with the SANRA (Scale for the Assessment of Narrative Review Articles) guideline and it is provided as Supplementary File 1.

### 2.7 Ethical Considerations

This study used data from publicly available published academic literature. No human participants were directly involved in this study. Therefore, no formal ethical clearance was sought to conduct the review.

## 3 Results

### 3.1 Distribution of Studies

As shown in Table 1, a total of 58 studies published from eight South Asian countries between 1980 and 2025 were included in this narrative review [11, 12, 13, 14, 15, 16, 17, 18, 19, 20, 21, 22, 23, 24, 25, 26, 27, 28, 29, 30, 31, 32, 33, 34, 35, 36, 37, 38, 39, 40, 41, 42, 43, 44, 45, 46, 47, 48, 49, 50, 51, 52, 53, 54, 55, 56, 57, 58, 59, 60, 61, 62, 63, 64, 65, 66, 67, 68]. The majority were conducted in India (*n* = 32, 55.1%) [11, 13, 14, 16, 19, 20, 21, 22, 23, 24, 25, 26, 27, 28, 29, 30, 31, 32, 33, 34, 35, 36, 37, 38, 39, 40, 41, 42, 43, 44, 45, 46, 47], followed by Pakistan (*n* = 17, 29.3%) [12, 15, 16, 52, 53, 54, 55, 56, 57, 58, 59, 60, 61, 62, 63, 64, 65], Nepal (*n* = 4, 6.9%) [48, 49, 50, 51], Bangladesh (*n* = 3, 5.2%) [17, 18, 68], and Sri Lanka (*n* = 2,3.4%) [66, 67]. No study was identified from Afghanistan, Bhutan, and the Maldives. Sample sizes ranged from 29 to 909 [49, 55]. Most studies were cross‐sectional studies (*n* = 54, 93.1%); nonetheless, there were also prospective, cohort, and longitudinal studies. Most studies were conducted among medical students (*n* = 40, 68.9%) [12, 13, 16, 17, 18, 20, 21, 22, 23, 27, 28, 29, 30, 31, 32, 34, 35, 37, 40, 41, 43, 45, 46, 49, 51, 52, 53, 54, 55, 56, 57, 58, 59, 62, 63, 64, 65, 66, 67], a smaller proportion studied among physicians (*n* = 11, 18.9%) [11, 14, 15, 33, 36, 38, 39, 42, 47, 50, 61] and mixed populations (*n* = 7, 12.1%) [19, 24, 27, 44, 48, 60, 68].

**Table 1:** Domains and factors assessed in studies on medical students' and physicians' attitudes toward psychiatry and career choice in South Asia.

SN	Study	Country	Sample size	Type of Study	Domain assessed	Key findings
1	Giasuddin et al., 2015 [17]	Bangladesh	200	Cross sectional	Mental health stigma & ATP	Fifth‐year students had higher stigma toward persons with mental disorders than first‐year students (*p *= 0.028). Attitudes toward psychiatry were slightly more favorable in fifth‐year students (*p *= 0.073) and female students (*p *= 0.018).
2	Sarker et al., 2017 [18]	Bangladesh	152	Cross sectional	ATP	Only 2.6% of 5th‐year students wanted to specialize in psychiatry. A higher proportion of 5th‐year students (74%) compared to 1st‐year students (40%) agreed psychiatrists earn as much as other physicians.
3	Mujahid et al., 2024 [18]	Bangladesh	640	Cross sectional	Career choice preferences	Psychiatry ranked 9th overall as a specialty choice (17.8%). Career choice was heavily influenced by family (67.8%), friends (49.5%), practicing physicians (54.8%), and senior colleagues (45.8%).
4	Jilowa et al., 2018 [19]	India	206	Cross sectional	ATP	Second‐year students had more positive overall attitudes toward psychiatry than interns (83% vs. 48.1%). Mean ATP‐30 scores: 99.7 for second‐year students versus 94.5 for interns. 10% of second‐years and 6.6% of interns considered psychiatry as a career.
5	Chawla et al., 2012 [20]	India	164	Cross sectional	ATP and career preference	Only 39.6% had considered psychiatry as a career, 47.6% rejected it, and 12.8% were unsure. Reasons for considering psychiatry: challenging (73.8%) and interesting (46.2%). Reasons for rejecting psychiatry: difficult patient management (51.3%), uncertainty about the field (26.9%), family pressure (17.9%), and fear of acquiring psychiatric illness (16.7%).
6	Mukhim et al., 2025 [21]	India	102	Cohort	ATP and mental illness	Before exposed to psychiatry – Positive ATP‐30 was −102.2(Positive); After exposed to psychiatry – ATP‐30 was − 105.7 (Positive) Before exposed to psychiatry − 21.4% preferred psychiatry as their career After exposed to psychiatry − 18.4% preferred psychiatry as their career.
7	Srivastava, 2012 [22]	India	62	Cross sectional	ATP	Attitude was negative, only 1 (1.5%) preferred psychiatry as a career; 2 (3%) considered it a possibility; 71.5% rejected psychiatry.
8	Aruna et al., 2016 [23]	India	404	Cross sectional	ATP	Only 25% of students expressed willingness to pursue psychiatry as a career, while 50.9% ruled it out. Knowledge and attitudes improved with advancing medical year: correct responses increased from 31.9% in the 1st year to 57.5% in the final year.
9	Gulati et al., 2014 [24]	India	135	Cross sectional	AMI	Exposure to psychiatry during clinical rotations was linked with positive attitudes toward psychiatry.
10	Konwar et al., 2012 [25]	India	110	Cross sectional	ATP & AMI	Students exposed to psychiatry postings (37%) demonstrated more favorable attitudes across all domains compared to those without exposure (63%). Before exposed to psychiatry, 37% of students preferred psychiatry as their career, and after exposed to psychiatry, it was increased to 51%.
11	Tharyan et al., 2001 [26]	India	247	Cross sectional	ATP & AMI	ATP scores were positive across groups however did not significantly improve with exposure to psychiatry. 20.6% preferred psychiatry as a career; 39.3% rejected psychiatry.
12	Lingeswaran., 2010 [27]	India	480	Cross sectional	ATP & AMI	Psychiatry is the least preferred career choice. Many agreed psychiatry has low prestige among the public (up to 85% among interns).
13	Praharaj et al., 2013 [28]	India	67	Cross sectional	ATP & AMI	34.8% preferred psychiatry as a career (ranked 8th); 65.2% rejected it. Misconceptions persisted: 50% had reservations about ECT, some fearing permanent brain damage.
14	Alexander and Kumaraswamy, 1995 [29]	India	146	Cross sectional	ATP & AMI	Psychiatric clinical postings: 67% reported positive influence; however, only 6 (4.1%) preferred psychiatry as a career.
15	Kumar and Dhaliwal., 2010 [30]	India	282	Cross sectional	Specialty choice	Only 6 (5.1%) preferred psychiatry as a career: Male‐ 5 & Female‐ 1. 2.3% of 2nd semester students preferred psychiatry as a career & the preference rose slightly to 6.1% in the 6th semester and 6.1% in the 8th semester.
16	Rajagopalan and Kuruvilla, 1994 [31]	India	30	Cross sectional	ATP & AMI	Both pre‐posting & post‐posting attitudes were positive (no statistically significant improvement after 2‐week posting)
17	Subba et al., 2012 [32]	India	373	Cross sectional	Specialty choice	19.3% considered psychiatry as a career (overall). Gender distribution in psychiatry: 30 male (41.7%), 42 female (58.3%).
18	Kodakandla et al., 2016 [33]	India	176	Cross sectional	ATP & AMI	23 (13%) preferred psychiatry as a career; 40% said they would “consider psychiatry” if other choices were unavailable.
19	Desai et al., 2019 [13]	India	143	Longitudinal	ATP & AMI	Before exposure ATP‐ 89.83, after exposure ATP‐ 93.88 (significant). Before exposed to psychiatry 27.97% student preferred psychiatry as their career and after exposed to psychiatry, it increased to 49.65%.
20	Alex et al., 2025 [14]	India	100	Cross sectional	ATP & AMI	Before exposure ATP‐ 109.47 ± 10.51, after exposure ATP‐115.12 ± 12.50 (Significant). Before exposed to psychiatry − 17% preferred psychiatry as their career, after exposed to psychiatry it increased to 31%.
21	Desai and Chavda, 2018 [34]	India	116	Cross sectional	ATP & AMI	Median scores: AMI = 54/100 (neutral), ATP = 82/150 (neutral). 63% preferred psychiatry as their career.
22	Dinesan et al., 2020 [11]	India	100	Cross sectional	ATP & AMI	Before exposure ATP‐ 90.2, after exposure ATP‐120.4. Before exposed to psychiatry − 16% preferred psychiatry as their career; after exposed to psychiatry it increased to 23%.
23	Jahagirdar, 2022 [35]	India	295	Cross sectional	ATP & AMI	19.4% preferred psychiatry as their career. 47.1% excluded psychiatry from “most exciting specialties”; 47.5% felt psychiatrists are less respected; 50% said psychiatrists earn less; 43.7% believed most psychiatric patients are violent.
24	Netto et al., 2018 [36]	India	148	Cross sectional	ATP	Overall attitude score: Males = 88.40, Females = 85.25 (*p* < 0.001, more positive in males). 3.2% of males (*n* = 3) and 0% of females preferred psychiatry as a career.
25	Jain et al., 2014 [37]	India	250	Cross sectional	ATP	Only 28 (11.2%) preferred psychiatry as their career and 123 (49.2%) rejected psychiatry outright. However, foreign students were more favorable toward psychiatry than Indian students.
26	Garg et al., 2020 [38]	India	98	Cross sectional	ATP	Before exposure ATP‐ 108.0, after exposure ATP‐113.3. Before exposure 18 (18.4%) interns preferred psychiatry as their career; after exposure it increased to 33 (33.7%) interns.
27	Sharma et al., 2018 [39]	India	93	Prospective	ATP	Before exposure ATP‐ 107, after exposure ATP‐ 106 (Not significant)
28	Godasi et al., 2021 [40]	India	596	Cross sectional	ATP & AMI	Only 18.8% willing to take psychiatry; 51.5% would choose psychiatry if no other specialty were available. 11.1% felt family would be horrified if they chose psychiatry.
29	Dey et al., 2019 [41]	India	104	Cross sectional	ATP	Overall negative attitudes toward psychiatry as a discipline and career was found. Females scored slightly higher (81.9) than males (80.1).
30	Prasad et al., 2016 [42]	India	44	Cross sectional	ATP	Positive attitude to psychiatry. All 29 who completed psychiatry postings found them useful; 15 wanted longer postings.
31	Rao et al., 1989 [43]	India	50	Prospective	ATP	Before exposure: 54% against psychiatry; no (0%) female student considered psychiatry as a career; 5 males (16%) were interested (only 2 definite). After exposure: slight positive shift 16% of males showed interest, 2 females expressed interest in child psychiatry/psychology; “Definite No” responses decreased from 34% to 25%.
32	Bhise & Kochar, 2020 [44]	India	300	Cross sectional	ATP	All groups had overall positive attitudes Undergraduates − 94.4, interns − 94.6, postgraduates − 116.2, Answer for “I would like to be a psychiatrist” had a mean score of 2.22 for the final year, 2.91 for interns, and 2.91 for postgrads.
33	Kumar et al., 2024 [45]	India	101	Cross sectional	ATP	82% found posting duration adequate, 83%–84% were satisfied with the teaching pattern and content; however, but only 37% preferred psychiatry as a career and 59% “may consider” it.
34	Behere et al., 2021[46]	India	Not specified	Cross sectional	ATP	The improvement in attitude & knowledge in male medical students is more than 3 times of that seen in female medical students, after two weeks of training.
35	Raikot et al., 2023 [47]	India	203	Cross sectional	ATP	Overall attitude: Positive (mean ATP‐30 = 109.3). Females had significantly more positive attitudes than males (111 vs. 107, *p* = 0.024). Before exposure 16% preferred psychiatry as a career and after exposure 33% preferred psychiatry as a career.
36	Risal et al., 2013 [48]	Nepal	159	Cross sectional	ATP & AMI	The majority of responses were positive or neutral on both ATP‐30 and AMI. Answer for “I would like to be a psychiatrist” had a mean score for male were 2.6, for female were 2.4 (*p* < 0.05), for students were 3.1, for interns were 2.2.
37	Chadda and Singh, 1999 [49]	Nepal	29	Cross sectional	ATP & AMI	Before exposure, 13.8% of participants preferred psychiatry as their career, after exposure it increased to 17.9%. Awareness of psychological treatment methods rose sharply from 34.5% to 96.4%.
38	Shakya, 2018 [50]	Nepal	50	Cross sectional	ATP	12 (24%) participants preferred psychiatry as their career
39	Adhikari, 2017 [51]	Nepal	280	Cross sectional	ATP & AMI	Participants showed broadly positive attitudes towards psychiatry and mentally ill patients. Initially psychiatry was among the top three choices for 15 first‐year students, but this number declined to 8 in the third year and 4 in the fourth year. At the time of the survey, psychiatry preference increased, reported by 11 first‐year, 8 third‐year, and 9 fourth‐year students.
40	Khan et al., 2008 [52]	Pakistan	281	Cross sectional	ATP	Both male and female students showed positive attitude. Answer for “I would like to be a psychiatrist” mean score for male were 2.3, for female were 2.6.
41	Niaz et al., 2003 [53]	Pakistan	281	Cross sectional	ATP	Nearly more than 60% students hold positive views about psychiatry. 54% of students were reluctant to choose psychiatry as a career, citing financial concerns, social respect, and uncertainty about the field's future in Pakistan.
42	Rehman et al., 2011 [54]	Pakistan	771	Cross sectional	Specialty preferences	13.1% participants preferred psychiatry as their career. The highly considered factors (regarding specialties) chosen by 70% of the medical students were personalities of the individuals, prestige and respect, international opportunities, and time commitment.
43	Syed et al., 2008 [16]	Pakistan	229	Cross sectional	ATP	More than 60% of the participants had a negative view of psychiatry. 7.6% participants preferred psychiatry as their career.
44	Aslam et al., 2009 [55]	Pakistan	909	Cross sectional	ATP	17% of participants preferred psychiatry as their career. Interest towards psychiatry was significantly higher among students in private institutes compared to public (24% vs. 14%, *p* = 0.001) and among exposed compared to non exposed (22% vs. 14%).
45	Sajid et al., 2009 [56]	Pakistan	89	Cross sectional	ATP	Awareness of the importance of psychiatric skills for all medical practitioners increased from 60% pre‐clerkship to 100% post‐clerkship. Before exposure 16 (24%) participants preferred psychiatry as their career, after exposure it decreased to 11 (23%)
46	Kareem et al., 2020 [57]	Pakistan	384	Cross sectional	ATP	Only 19% agreed psychiatry was among the three most exciting specialties; most students were reluctant to choose psychiatry as a career.
47	Fatima et al., 2021 [58]	Pakistan	831	Cross sectional	ATP	The most participants had a positive attitude towards psychiatry. Female, older participants and having any sort of exposure toward psychiatry had more positive attitude.
48	Awais et al., 2019 [59]	Pakistan	381	Cross sectional	ATP	10.2% of students ranked psychiatry as their top career choice; 24.2% considered it a possible choice. Females (11.1%) were slightly more inclined towards psychiatry compared to male (7.8%).
49	Ahmed et al., 2021 [60]	Pakistan	41	Cross sectional	ATP	Mean ATP‐30 score increased from 119 to 128 after exposure.
50	Haider, 2023 [61]	Pakistan	40	Cross sectional	ATP	Before exposure, 15% of participants preferred psychiatry as their career, after exposure it increased to 57.5%. After exposure there was significant improvement in knowledge, attitudes, and perceptions (52.2 vs 60.6).
51	Akhunzada et al., 2014 [62]	Pakistan	184	Cross sectional	ATP	Answer for “I would like to be a psychiatrist” before exposure mean score was 3.9 and after exposure mean score was 2.9.
52	Rahman and Choudhary, 2021 [63]	Pakistan	413	Cross sectional	ATP	Attitude towards psychiatry was positive, Mean ATP‐30 score: 108 ± 9.1 (range 88–129). Students with a first‐degree relative suffering from psychiatric illness had significantly higher ATP‐30 scores (117 vs. 102, *p* = 0.001).
53	Maqsood et al., 2006 [64]	Pakistan	538	Cross sectional	ATP	Answer for “I would like to be a psychiatrist” mean score for male was 2.6 and mean score for female was 2.9.
54	Khokhar et al., 2014 [12]	Pakistan	107	Cross sectional	ATP	Before exposure, both groups had comparable ATP‐30 scores (Study = 84.5; Control = 84.6; *p* > 0.93). After exposure, the study group showed a significant increase in total ATP‐30 scores (103.1 vs 85.8; *p* < 0.001).
55	Khalid et al., 2018 [65]	Pakistan	212	Cross sectional	ATP	Before exposure, ATP‐30 scores (Male = 98.8; Female = 105.9). After exposure, ATP‐30 scores (Male = 100.5; Female = 105.1).
56	Asif et al., 2014 [15]	Pakistan	401	Cross sectional	Speciality preference	Only 7 (1.7%) participants preferred psychiatry as their career.
57	Rodrigo et al., 2012 [66]	Sri Lanka	93	Cross sectional	ATP	Before exposure, ATP‐30 scores 100 ± (positive), after exposure, ATP‐30 scores 110.8 (positive). Before exposure, 9.4% of participants preferred psychiatry as their career; after exposure it increased to 25.8%.
58	Baminiwatta et al., 2020 [67]	Sri Lanka	711	Cross sectional	ATP	92.2% showed an overall “positive” attitude, and the ATP score was 107.7. 22.2% of participants preferred psychiatry as their career.

Abbreviations: AMI, attitudes towards mental illness; ATP, attitudes toward psychiatry.

The first study that we could find about the attitudes towards psychiatry in South Asia was published in 1989 [43]; however, most studies were published in the 21st century. Most studies on attitudes towards psychiatry among medical students and physicians were published between 2010 and 2019 (*n* = 30) [13, 17, 19, 20, 22, 23, 24, 25, 27, 28, 30, 32, 33, 34, 36, 37, 39, 41, 48, 50, 51], followed by an increasing number in the most recent period, 2020–2025 (*n* = 17) [11, 14, 21, 35, 38, 44, 46, 47, 57, 58, 60, 61, 63, 67]. This pattern indicates that research interest in psychiatric stigma and professional attitudes seems to have gained importance in the past 15 years.

### 3.2 Methods of Assessing Attitude

Most studies used *Attitudes Toward Psychiatry* (ATP‐30) [11, 12, 13, 14, 19, 21, 23, 26, 34, 35, 38, 39, 41, 44, 46, 47, 48, 50, 52, 57, 58, 60, 62, 63, 64, 65, 66, 67], ATP‐29 [24, 27, 33], *Balon's Questionnaire* [36, 42], *Attitudes towards Mental Illness* (AMI) [21, 26, 27, 34, 48], and other questionnaires [16, 18, 20, 22, 23, 27, 29, 31, 40, 43, 45, 53, 61, 68]; however, some studies used self‐developed questionnaires (*n* = 23) (Table 2). The most widely used method was the ATP‐30, a 30‐item questionnaire developed by Burra et al. in 1982 [69] to measure attitudes of medical students and physicians toward psychiatry, mainly in response to concerns about stigma and declining interest in psychiatry. It uses a 5‐point Likert scale and covers domains such as the overall merits of psychiatry, the role of psychiatrists, the efficacy of psychiatric treatment, and psychiatry as a career choice. The ATP‐30 has been shown to have good reliability and validity, making it one of the most frequently used methods to measure attitudes towards psychiatry; nonetheless, the original validation study showed that the ATP‐30 primarily measures a single overall attitude toward psychiatry [69].

**Table 2:** Attitude towards Psychiatry.

SN	Study	Country	Population	Tool used	Attitude towards psychiatry
1	Giasuddin et al., 2015 [17]	Bangladesh	Student	1. MCAQ 2. AQ‐27 3. MBAQ	Female, upper medical school year, and middle socioeconomic level were more positive
2	Sarker et al., 2017 [18]	Bangladesh	Student	Self‐developed Questionnaire	Both 1st and 5th year students were positive but majority of students were not considering psychiatry as a career.
3	Mujahid et al., 2024 [68]	Bangladesh	Mixed	Self‐developed Questionnaire	Positive (but far less attractive compared to clinical specialties).
4	Jilowa et al., 2018 [19]	India	Mixed	ATP‐30	Positive (Score of ATP for Students were 99.7 and for Interns were 94.5)
5	Chawla et al., 2012 [20]	India	Student	Self‐developed Questionnaire	Positive (39.6% of students had considered psychiatry as a career)
6	Mukhim et al., 2025 [21]	India	Student	1. ATP‐30 2. AMI	Before exposed to psychiatry – Positive (ATP: 102.19) After exposed to psychiatry – Positive (ATP: 105.65)
7	Srivastava, 2012 [22]	India	Student	Self‐developed Questionnaire	Negative (1.5% of students had considered psychiatry as a career)
8	Aruna et al., 2016 [23]	India	Student	Self‐developed Questionnaire	Positive (25% of students had considered psychiatry as a career)
9	Gulati et al., 2014 [24]	India	Mixed	1. ATP‐29 scale 2. OMI scale	Positive (Exposure to psychiatry during clinical rotations was linked with positive attitudes toward psychiatry and mental illness)
10	Konwar et al., 2012 [25]	India	Student	ATP‐30 scale	Positive (Before being exposed to psychiatry, 37% of students preferred psychiatry as their career, and after being exposed to psychiatry, it increased to 51%.)
11	Tharyan et al., 2001 [26]	India	Mixed	1. ATP‐30 2. AMI	Before exposed to psychiatry ‐ Positive (Score of ATP: 107.2) After exposed to psychiatry ‐ Positive (Score of ATP: 107.5)
12	Lingeswaran., 2010 [27]	India	Student	1. ATP‐29 2. AMI	Negative (Psychiatry was the least preferred career choice)
13	Praharaj et al., 2013 [28]		Student	Self‐developed Questionnaire	Positive (34.8% of students had considered psychiatry as a career)
14	Alexander and Kumaraswamy, 1995 [29]	India	Students	Self‐developed Questionnaire	Positive (67% reported positive influence after clinical posting)
16	Rajagopalan and Kuruvilla, 1994 [30]	India	Student	Self‐developed Questionnaire	Before exposed to psychiatry ‐ Positive After exposed to psychiatry ‐ Positive (Not Significant)
17	Kodakandla et al., 2016 [33]	India	Physician	ATP‐29 scale	Positive (13% of students had considered psychiatry as a career)
18	Desai et al., 2019 [13]	India	Student	1. ATP‐30 2. MICA Scale	Before exposed to psychiatry – Neutral (ATP: 89.8); After exposed to psychiatry – Positive (ATP: 93.9)
19	Alex et al., 2025 [14]	India	Physician	ATP‐30	Before exposed to psychiatry ‐ Positive (ATP:109.5) After exposed to psychiatry ‐ Positive (ATP:115.1)
20	Desai and Chavda., 2018 [34]	India	Student	1. ATP‐30 2. AMI‐20 Scale	Neutral (Score of ATP: 82 Median scores of AMI: 54)
21	Dinesan et al., 2020 [11]	India	Physician	ATP‐30	Before exposed to psychiatry – Neutral (ATP: 90.2) After exposed to psychiatry – Positive (ATP: 120.3)
22	Jahagirdar, 2022 [35]	India	Student	ATP‐30	Positive (19.4% of students had considered psychiatry as a career)
23	Netto et al., 2018 [36]	India	Physician	29‐item Balon's questionnaire	Neutral (ATP: Male‐88.40; Female‐85.25).
24	Garg et al., 2020 [38]	India	Physician	ATP‐30	Before exposed to psychiatry – Positive (ATP: 108.0) After exposed to psychiatry ‐ Positive (ATP: 113.3)
25	Sharma et al., 2018 [39]	India	Physician	ATP‐30	Before exposed to psychiatry – Positive (ATP: 107) After exposed to psychiatry – Positive (ATP: 106) (Not Significant)
26	Godasi et al., 2021 [40]	India	Student	1. BTMI 2. Self‐developed Questionnaire	Negative (11.1% felt family would be horrified if they chose psychiatry)
27	Dey et al., 2019 [41]	India	Student	ATP‐30	Negative (ATP: 80.6)
28	Prasad et al., 2016 [42]	India	Physician	Balon's questionnaire	Before exposed to psychiatry ‐ Positive After exposed to psychiatry – Positive (All 29 who completed psychiatry postings found them useful; 15 wanted longer postings.)
29	Rao et al., 1989 [43]	India	Student	ATP	Positive (psychiatry training improved perceptions and reduced stigma and rejection but did not increase much in career choice)
30	Bhise and Kochar., 2020 [44]	India	Mixed	ATP‐30	Positive (ATP for Final years were 94.4; for Interns were 94.5; for Postgraduates were 116.2)
31	Kumar et al., 2024 [45]	India	Student	Self‐developed Questionnaire	Positive (99% reported positive change in attitudes toward Persons with Mental Illness
32	Behere et al., 2021[46]	India	Student	ATP‐30	After exposed to psychiatry ‐Positive (Male has 3 times more improvement than Female)
33	Raikot et al., 2023 [47]	India	Physician	ATP‐30	Positive (ATP‐30: 109.2).
34	Risal et al., 2013 [48]	Nepal	Mixed	1. ATP‐30 2. AMI‐20 Scale	Neutral (Most of the question of ATP‐30 and AMI had neutral mean score)
35	Shakya, 2018 [49]	Nepal	Physician	ATP‐30	Positive (84% viewed psychiatry as a rapidly advancing subject)
36	Khan et al., 2008 [52]	Pakistan	Student	ATP‐30	Positive (Both male and female students showed positive attitude towards most of the items except few, towards which the attitude of the students was neutral.)
37	Niaz et al., 2003 [53]	Pakistan	Student	Self‐developed Questionnaire	Positive (Nearly more than 60% students hold positive views about psychiatry)
38	Syed et al., 2008 [16]	Pakistan	Student	Self‐developed questionnaire	Negative (More than 60% of the participants had a negative view of psychiatry)
39	Sajid et al., 2009 [56]	Pakistan	Student	Self‐developed Questionnaire	Positive (More students found psychiatric patients interesting after the clerkship from 55% to 66%)
40	Kareem et al., 2020 [57]	Pakistan	Student	ATP‐30	Positive (82% agreed psychiatry should be an important part of the medical curriculum)
41	Fatima et al., 2021 [58]	Pakistan	Student	ATP‐30	Positive (ATP: 107.6)
42	Ahmed et al., 2021 [60]	Pakistan	Mixed	ATP‐30	Before exposure to psychiatry: Positive (ATP: 119) After exposure to psychiatry: Positive (ATP: 128)
43	Haider, 2023 [61]	Pakistan	Physician	Self‐developed Questionnaire	Before exposed to psychiatry‐ Positive (ATP: 52.2; After exposed to psychiatry‐ Positive (ATP: 60.6) also Before exposure 15% participants considered psychiatry as their career, after exposure it increased to 57.5%
44	Akhunzada et al., 2014 [62]	Pakistan	Student	ATP‐30	Positive (Most of the question of ATP‐30 had positive mean score)
45	Rahman and Choudhary, 2021 [63]	Pakistan	Student	ATP‐30	Positive (ATP: 108)
46	Maqsood et al., 2006 [64]	Pakistan	Student	ATP‐30	Positive (mean score for male was 2.65 and mean score for female was 2.86)
47	Khokhar et al., 2014 [12]	Pakistan	Student	ATP‐30	Before exposed to psychiatry – Negative (ATP: 84.5) After exposed to psychiatry – Positive (ATP: 103.1)
48	Khalid et al., 2018 [65]	Pakistan	Student	ATP‐30	Before exposed to psychiatry – Positive (ATP: 104.5) After exposed to psychiatry ‐Positive (ATP: 104.2)
49	Rodrigo et al., 2012 [66]	Sri Lanka	Student	ATP‐30	Before exposed to psychiatry – Positive (ATP: 100) After exposed to psychiatry – Positive (ATP: 110)
50	Baminiwatta et al., 2020 [67]	Sri Lanka	Student	ATP‐30	Positive (ATP: 107.8)

Abbreviations: AMI, Attitudes towards Mental Illness scale; AQ‐27, Attribution Questionnaire‐27; ATP, Attitudes Toward Psychiatry; BTMI, Beliefs Toward Mental Illness scale; GHQ, General Health Questionnaire; MBAQ, Modified Balon Attitudes Questionnaire; MCAQ, Modified Corrigan Attribution Questionnaire; MICA, Mental Illness Clinicians' Attitudes scale; OMI, Opinions about Mental Illness scale; VAS, Visual Analogue Scale.

A shorter version, the ATP‐29, was also used. This scale measures attitudes toward psychiatry by using a four‐point Likert‐type self‐rated scale. It covers domains such as attitude to psychiatric patients, psychiatrists, psychiatric institutions, illness and treatment, knowledge, and career choice [24].

In addition, some studies employed the *Balon's Questionnaire*, a 29‐point questionnaire with a 4‐point Likert scale specifically designed to assess medical students' career preferences in psychiatry and related specialties [70].

Non‐validated semi‐structured questionnaires that were specifically developed for the sociocultural and educational context of South Asian medical students and physicians were used in 23 studies. These instruments were designed to capture relevant perceptions, cultural beliefs, and professional concerns that may not be adequately addressed in validated international methods. Although these questionnaires may provide context‐specific information, they limit comparability across studies because of their lack of validation.

### 3.3 Status of Attitudes towards Psychiatry and Choosing Psychiatry as a Career

Most studies indicate that students and physicians have a positive attitude (*n* = 41) towards psychiatry, and only in five studies found physicians or students have a negative attitude [4, 16, 22, 27, 41]. In some studies attitude was not mentioned or found neutral (Tables 1, 2).

**Figure 1:**
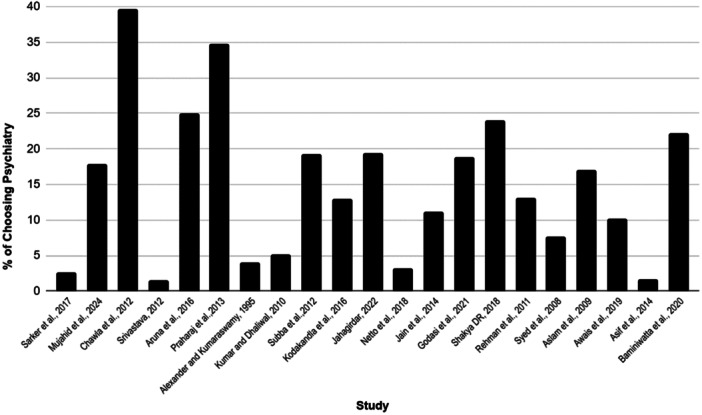
Percentage of medical students and physicians choosing psychiatry as a career in South Asia.

Although positive attitude is higher than negative, only a small percentage of participants agreed that they want to become psychiatrist, typically ranging from 5% to 25% (Figure 1). Even in some studies, psychiatry was one of the least preferred choices as a career option [15, 27, 30].

In total, 28 studies assessed changes in attitudes or career choice before and after exposure to psychiatry. Of these, 20 studies reported that attitude or career choice towards psychiatry increased among students or physicians after their exposure to psychiatry [11, 12, 13, 14, 24, 25, 30, 38, 42, 43, 44, 45, 46, 47, 49, 53, 55, 60, 66], 5 studies [26, 31, 37, 39, 65] attitude remained the same; in one study [62] it showed negative, and in two studies, positive attitude towards psychiatry had increased, but their preference for psychiatry as a career decreased [21, 56].

**Figure 2:**
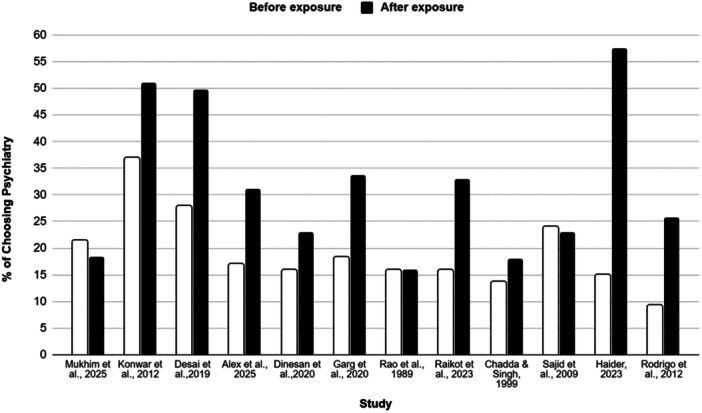
Impact of psychiatry exposure on career choice among medical students and physicians in South Asia.

In Bangladesh, Sarker et al. (2017) [18] showed favorable perceptions but very low rates of psychiatry as a career choice (0%–2.6%), but more recent findings from Mujahid et al. (2024) [68] showed some improvement, with 17.8% of participants considering psychiatry. In India, a positive attitude was reported in many studies, but the proportion selecting psychiatry as a career ranged widely from as low as 2.1% [30] to 63% [34]. In Nepal, attitudes towards psychiatry were mixed; Risal et al. (2013) [47] reported neutral views, while Shakya (2018) [50] reported positive attitudes. On the other hand, Chadda and Singh (1999) [49] reported increased career choice following exposure to psychiatry. Studies that were conducted in Pakistan also had positive attitudes. Niaz et al. (2003) [53] reported an increase from 20.5% to 25.5%, Aslam et al. (2009) [55] from 14% to 22%, and Haider (2023) [61] from 15% to 57.5% after clinical rotation. Khokhar et al. (2014) found a drastic change from negative (mean ATP‐30 score 84.5) to positive (103.1) following exposure [12]. However, Sajid et al. (2009) reported a reduction in career choice from 24% to 23% [56]. In Sri Lanka, Rodrigo et al. (2012) observed a notable increase in career preference after exposure (9.4% to 25.8%), which is similar to the rest of the studies (Figure 2) [66].

### 3.4 Associated Factors

The studies examined a wide range of demographic, educational, and psychosocial factors that can influence attitudes towards psychiatry. Commonly investigated factors included gender, prior exposure to psychiatry, family history of mental illness, type of medical institution (government vs. private), attitude of society and fellow medical professionals towards psychiatry, economic opportunities, and cultural or religious influences on career choice. 28 studies also assessed the impact of exposure by clinical postings/clerkships or lecture classes in psychiatry, where attitude towards psychiatry has been changed significantly in most of the studies [11, 12, 13, 14, 21, 24, 25, 26, 29, 31, 37, 38, 39, 42, 43, 44, 45, 46, 47, 49, 55, 56, 58, 60, 61, 62, 65, 66]. In some studies, comparisons were made between psychiatry and other specialties to highlight relative preferences, where psychiatry was always the least preferred subject [15, 27, 30].

16 studies reported differences in attitude towards psychiatry between male and female [17, 26, 30, 36, 40, 43, 46, 47, 48, 51, 52, 58, 59, 63, 64, 65] and in one study both male and female had similar attitude (mean ATP male = 82; female ATP = 83, *p* > 0.05) [28]. 11 studies out of 16 reported female students or physicians had more positive attitudes and interest towards psychiatry as a career choice [17, 26, 40, 41, 47, 52, 58, 59, 63, 64, 65].

Attitudes tended to become more favorable in recent studies in comparison to earlier studies, which was consistent in all the countries [14, 15, 18, 34, 49, 50, 61, 68] except in Sri Lanka, where in a recent study the percentage of interest towards psychiatry had been reduced [66, 67].

## 4 Discussion

### 4.1 Overall Attitudes Toward Psychiatry

This narrative review of 58 studies across South Asia provides a comprehensive picture of medical students' and physicians' attitudes toward psychiatry. Overall, the findings suggest that attitudes are predominantly positive, nonetheless most of the students or physicians do not prefer psychiatry as their profession. Similar findings were found in a systematic review of 32 studies across 22 countries [8].

### 4.2 Impact of Exposure to Psychiatry

A key finding is the **i**mpact of exposure to psychiatry. Multiple studies from India, Pakistan, and Sri Lanka showed that attitudes improved significantly following psychiatric postings/clinical rotations, or seminars, which is also similar to the previous systematic review finding [8]. For example, in India, Alex et al. (2025) and Dinesan et al. (2020) demonstrated measurable improvement in ATP scores after exposure [11, 14], while in Pakistan, Khokhar et al. (2014) reported a shift from negative to positive perceptions after training [12]. Similar findings were documented in Sri Lanka [66]. Exposure to psychiatry not only positively shifts attitudes toward psychiatry but also increases the proportion of individuals choosing it as a career. For example, in India, Desai et al. (2019) and Garg et al. (2020) reported that the proportion of individuals choosing psychiatry as a career almost doubled after psychiatry exposure [13, 38]. In Pakistan, the proportion almost increased four times [61], and in Sri Lanka it almost tripled [66]. Similar results were found in the UK, where 14.9% of physicians with psychiatry exposure chose psychiatry careers compared to only 1.8% without exposure [71]. Similarly, Adekunte et al. (2016) showed that after a 4‐week psychiatry placement, the percentage of students choosing psychiatry among their top three career choices increased from 7% to 20% [72]. These results from our study and other global studies indicate that structured psychiatry education, training, and direct exposure can reduce misconceptions and stigma and increase interest in psychiatry as a career.

### 4.3 Negative Attitudes

Although most studies reported positive attitudes towards psychiatry, negative or neutral attitudes can be seen in various studies. Some studies [21, 24, 46] conducted in India reported negative attitudes because the participants had perceptions of psychiatry as less scientific, less prestigious, or associated with limited treatment outcomes. In Pakistan, Syed et al. (2008) reported more than 60% negative responses [16]. Just like South Asian countries, Chinese medical students also had negative attitudes toward psychiatry due to many reasons, particularly regarding financial reward and prestige issues [73]. Similar responses were given by Italian and Spanish students [74, 75]. These results suggest that progress has been made, however psychiatrists still continue to face stigma, and it exists among both the general public and medical professionals [73].

### 4.4 Career Preference and Paradoxical Findings

Most of the studies reported positive attitudes, nonetheless career preference for psychiatry still remains low. Several studies reported that while students acknowledged psychiatry's importance, only a minority considered it as a career option [18, 19, 21, 29]. These paradoxical findings can also be seen in other international studies where attitude toward psychiatry is positive, however students and physicians are more interested in choosing other specialties as their careers instead of psychiatry. In Germany, despite positive expectations about psychiatric practice, only 17% of students expressed interest in psychiatry as a specialty [76]. Similarly, in Kenya, 75% of medical students had positive attitudes toward psychiatry, however only 14.3% considered it as a potential career choice, and 66% rejected it [77].

### 4.5 Gender Differences in Interest

Our narrative review revealed that the majority of studies reported that females are more interested in choosing psychiatry as their career [17, 26, 40, 41, 47, 52, 58, 59, 63, 64, 65]. In another study, it was reported that Australian female medical students also had a greater interest in psychiatry and preferred psychiatry as a career [78]. Similar results were found in the UK, however the differences in gender are reducing in modern times [76].

### 4.6 Impact of Social Media and the COVID‐19 Pandemic

Recent events such as the COVID‐19 pandemic and the rise of social media have affected how medical students and physicians view psychiatry. Social media can increase awareness of mental health issues and encourage interest in psychiatry [79]; however, it can also spread stigma or misinformation [80]. The COVID‐19 pandemic brought mental health into public discussion, making people more aware of its importance [81]. At the same time, it highlighted challenges for mental health professionals, such as stress and burnout [82]. These changes show that medical education should adapt by addressing both the positive and negative influences of social media and preparing students for the realities of practicing psychiatry during global crises. Additionally, media depicting reinforcing the enduring stigma towards psychiatry can negatively affect career preference among physicians.

### 4.7 Implications for Education and Policy

The findings of this narrative review reported several important patterns that can be implicated to improve psychiatric education and reduce stigma in South Asia. First, 20 out of 28 studies reported improved attitudes towards psychiatry following psychiatry postings, lectures, or clerkships, which shows the importance of structured and adequate clinical exposure in medical curricula [11, 12, 13, 14, 24, 25, 26, 29, 31, 37, 38, 39, 42, 43, 44, 45, 46, 47, 49, 55, 56, 58, 60, 61, 62, 65, 66]. Curriculum planners should therefore prioritize early and repeated exposure to psychiatry and ensure interactive teaching, case‐based discussions, and mentorship. A comparative review of undergraduate psychiatry curricula in Bangladesh, India, Nepal, and Sri Lanka found wide heterogeneity, and most centres rely on short time clinical postings and lecture‐based teaching [83]. Psychiatry has been considered as a different subject in undergraduate exam in Sri Lanka only, while it is considered as part of medicine in other countries i.e., Bangladesh, Nepal, and India [83]. Among Bangladesh, India, Nepal, and Sri Lanka, Sri Lanka has the highest duration of clinical placement, and Bangladesh has the lowest time allocation (3 weeks clinical rotation and 20 h dyadic lecture). The low number of psychiatrists, ongoing brain drain, and lack of psychiatry training in internship rotations leads to inadequate mental health treatment [84]. Both studies emphasize on clinical rotations for improving attitudes toward psychiatry [83, 84]. Given that most South Asian medical schools already operate under considerable resource and faculty constraints, extending clinical exposure need not require entirely new infrastructure; rather, existing short postings, particularly in low‐allocation settings and where a longer standalone posting is not feasible in the near term, psychiatry teaching could instead be embedded within existing internal medicine or community medicine rotations, allowing exposure to be strengthened without demanding additional curricular time or staffing that many institutions in the region do not have [84].

Second, although most studies indicated positive attitudes, psychiatry was consistently ranked among the least preferred career options because most studies reported participants think this specialty have difficult work environment, inadequate income, low prestige, stigma, and discouragement from family and friends [16, 22, 24, 27, 40, 41]. These issues cannot be solved by curriculum reform; it needs systemic interventions such as student‐friendly postgraduate training pathways, offering research opportunities, improving financial incentives, and raising the professional prestige of psychiatrists in the medical community [8].

Third, gender differences were evident, with 11 out of 16 studies showing female students more interested and positive towards psychiatry [17, 26, 40, 41, 47, 52, 58, 59, 63, 64, 65]. This suggests that targeted strategies to support and mentor interested female students could increase the number of psychiatrists. At the same time, further research is needed to understand why male students and physicians show less interest in psychiatry and how to solve this issue. Structured mentorship pairing interested students, particularly female students, with senior psychiatry residents or faculty is a comparatively low‐cost intervention that most training institutions in the region could implement without additional funding, and may be a practical starting point given the broader resource limitations discussed above [85].

Overall, the review highlights both progress and persistent challenges. The predominance of positive attitudes is encouraging, nevertheless the persistence of stigma and low career preference underscores the need for changing patterns of psychiatry education, promoting early psychiatric exposure, integrating psychosocial aspects into general medicine, and solving structural barriers in training and clinical practice. These can increase the number of students and physicians who will choose psychiatry as a profession.

### 4.8 Strengths and Limitations

To the best of our knowledge, this is the first review article of medical students' and physicians' attitudes toward psychiatry in South Asia, covering nearly five decades of research. The comparative perspective across different countries, populations, and exposure levels gave us valuable insights.

However, some limitations must be acknowledged. First, study selection was conducted primarily by a single reviewer rather than two independent reviewers, which may have introduced selection bias despite consultation with the lead author on uncertain cases. Second, there was considerable variation in tools and methodologies among studies. Most studies used standardized instruments like the ATP‐30, ATP‐29, Balon's Questionnaire, AMI, and other validated questionnaires, however, many studies used self‐developed unstructured questionnaires. Because no formal quality appraisal was performed, findings from these methodologically weaker studies are given equal narrative weight to those using validated instruments, which limited our scope to compare results. Third, many studies described attitudes and career choices without detailed statistical analysis, which restricted the cross‐study comparisons. Fourth, there was uneven representation of countries, with the majority of studies conducted in India and Pakistan and far fewer from Bangladesh, Nepal, and Sri Lanka, and none for Afghanistan, Bhutan and the Maldives. This imbalance likely reflects differences in research infrastructure, number of medical schools and psychiatry training capacity across countries rather than true differences in underlying attitudes. Consequently, findings drawn predominantly from India and Pakistan may not generalize to Afghanistan, Bhutan, or the Maldives from which no eligible studies were identified or fully to the comparatively under‐represented Bangladesh, Nepal, and Sri Lanka. Region‐wide conclusions in this review should therefore be interpreted cautiously and primary research from the under‐represented countries is needed before firm pan‐regional generalizations can be drawn. Finally, this review was restricted to studies published in English, which may have led to the exclusion of relevant studies published in local or regional languages.

## 5 Conclusion

This narrative review demonstrates that attitudes toward psychiatry among medical students and physicians in South Asia are generally positive, and they improved significantly after exposure to psychiatry training, clerkship, and lectures. However, career preference for psychiatry is still very much low compared to other specialties. So to develop the field of mental health and psychiatry, medical curricula should emphasize early and structured psychiatry exposure, mentorship, and training. Some institutional steps also need to be taken to enhance psychiatry's visibility, improve career pathways, and reduce systemic stigma. In the future, researchers should focus on longitudinal studies to find out the changes in attitudes and career preferences over time so that interventions to improve mental health services can be evaluated.

## Author Contributions

**S. M. Yasir Arafat:** conceptualization, supervision, writing – original draft, writing – review and editing, methodology. **Sadeed Hossain:** data curation, writing – original draft.

## Funding

The authors have nothing to report.

## Ethics Statement

This study used data from publicly available published academic literature. No human participants were directly involved in this study. Therefore, no formal ethical clearance was sought to conduct the review.

## Conflicts of Interest

Arafat, S.M. Yasir is an Editorial Board member of Health Science Reports. and a coauthor of this article. To minimize bias, they were excluded from all editorial decision‐making related to the acceptance of this article for publication.

## Transparency Statement

The lead author S.M. Yasir Arafat affirms that this manuscript is an honest, accurate, and transparent account of the study being reported; that no important aspects of the study have been omitted; and that any discrepancies from the study as planned (and, if relevant, registered) have been explained.

## Supporting information


Supporting File

## Data Availability

Data sharing not applicable to this article as no datasets were generated or analysed during the current study.
