# Supplementary material for: Attitudes and Career Preferences Towards Psychiatry in South Asia: A Narrative Review of Medical Student and Physician Perspectives
Source: Health Sci Rep. 2026 Aug 18;9(8):e73060. doi: 10.1002/hsr2.73060 (PMC13482348; doi:10.1002/hsr2.73060)
Supplement: Supplementary file 1 [file HSR2-9-e73060-s001.docx]

**Supplementary file 1: SANRA Checklist**

| **SANRA Item** | **Item Description** | **Page/Section in Manuscript** | **Score (0–2)** | **Justification** |
| --- | --- | --- | --- | --- |
| 1 | Justification of importance | Introduction (1^st^ & 2^nd^ para) | 2 | Introduction establishes the mental health burden in South Asia, the shortage of psychiatrists (<1 per 100,000 population), low mental health budget allocation, and the gap left by the prior systematic review (Lyons, 2013), justifying why this update is needed. |
| 2 | Statement of aims | Introduction (final para) | 2 | The aim is stated explicitly in both the Abstract and Introduction: to examine attitudes of medical students/physicians toward psychiatry in South Asia and identify factors influencing career preference. |
| 3 | Literature search description | Methods, "Search Strategy" | 1 | The manuscript reports the databases searched (PubMed, Scopus, Google Scholar), the search date (August 2, 2025), and the full Boolean search string, along with defined inclusion and exclusion criteria. However, the search and screening process falls short of current reporting expectations in three respects: (1) study selection was performed by a single author, with the second/lead author consulted only for "borderline or uncertain" cases rather than through independent dual screening, which introduces a risk of selection bias that the manuscript itself acknowledges as a limitation; (2) no PRISMA-style flow diagram or record count is provided showing the number of records identified, screened, and excluded at each stage — only the final included total (n = 58) is reported; (3) the review was restricted to English-language publications with no discussion of how many non-English studies were identified or what their exclusion might mean for the region-wide conclusions. |
| 4 | Referencing | Throughout | 2 | Reference list is extensive and well-matched to claims |
| 5 | Scientific reasoning | Discussion, "Strengths and Limitations" | 1 | The manuscript transparently discloses, in the Strengths and Limitations section, that no formal quality appraisal was conducted and that findings from studies using non-validated, self-developed questionnaires (23 of 58 studies) are given equal narrative weight to findings from studies using validated instruments (ATP-30, ATP-29, Balon's Questionnaire, AMI). While this disclosure reflects appropriate transparency, the synthesis itself (Results and Discussion) does not differentiate or weight findings according to instrument validity, so methodologically weaker and stronger studies contribute equally to the review's overall conclusions about attitudes and career preference. |
| 6 | Appropriate Presentation of data | Tables 1–2, Figs 1–2 | 2 | Tables 1 and 2 and Figures 1 and 2 present study-level data (country, sample size, tool used, attitude scores, career-preference percentages) that directly support the narrative synthesis |
|  | **Total** |  | **10/12** |  |
